# Atrial Septal Defect Surgical Closure Following Trametinib Utilization in Noonan Syndrome–Associated Hypertrophic Cardiomyopathy

**DOI:** 10.1016/j.jaccas.2026.107006

**Published:** 2026-02-20

**Authors:** Camden Hebson, Sydney Escott, Guillermo Beltran-Ale, Robert Dabal, Gregor Andelfinger, Kristin Linscott

**Affiliations:** a Division of Pediatric Cardiology, Department of Pediatrics, Heersink School of Medicine, University of Alabama at Birmingham, Birmingham, Alabama, USA; b Department of Pediatrics, Heersink School of Medicine, University of Alabama at Birmingham, Birmingham, Alabama, USA; c Division of Pediatric Pulmonology, Department of Pediatrics, Heersink School of Medicine, University of Alabama at Birmingham, Birmingham, Alabama, USA; d Division of Cardiothoracic Surgery, Department of Surgery, Heersink School of Medicine, University of Alabama at Birmingham, Birmingham, Alabama, USA; e CHU Sainte Justine, Department of Pediatrics, Université de Montréal, Montréal, Québec, Canada; f Department of Genetics, Heersink School of Medicine, University of Alabama at Birmingham, Birmingham, Alabama, USA

**Keywords:** atrial septal defect, cardiac surgery, hypertrophic cardiomyopathy, MEK inhibition, Noonan syndrome, RASopathy

## Abstract

**Background:**

Noonan syndrome–associated hypertrophic cardiomyopathy (NS-HCM) is often lethal when presenting severely in infancy, especially when associated with structural heart disease. Trametinib, an MEK inhibitor that attenuates abnormal signaling in the RAS/MAPK pathway, has been shown to improve NS-HCM outcomes. Surgery for associated heart defects in hypertrophic cardiomyopathy remains worrisome for increased risk of complications.

**First-in-Human/Early Reports Summary:**

An infant presented with a large secundum atrial septal defect complicating NS-HCM. The patient was treated with trametinib to improve cardiac hypertrophy and then underwent successful surgical closure of the atrial septal defect.

**Discussion:**

This serial strategy allowed for rescue from severe heart failure presentation to eventual discharge home without cardiac symptoms.

**Novelty:**

This is, to our knowledge, the first patient with NS-HCM to undergo medical treatment with trametinib and then cardiac surgery for a structural heart defect.

**Take-Home Messages:**

Infants with severe presentations of NS-HCM and congenital heart defects can be medically stabilized with trametinib and then undergo successful structural heart surgery.

## Graphical Abstract

**Figure:**
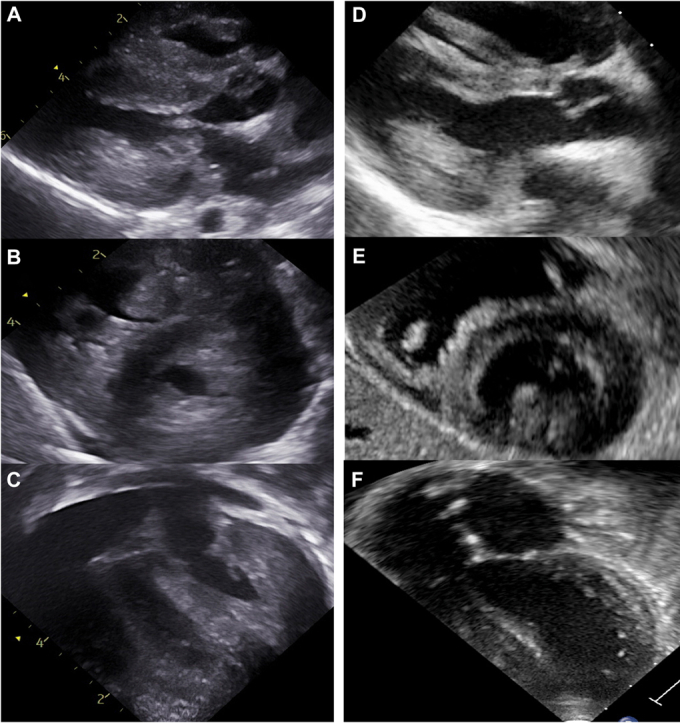

Early onset hypertrophic cardiomyopathy (HCM) related to Noonan syndrome has traditionally been associated with high mortality, with 60% of such infants dying from heart failure–associated complications within the first year of life.^1^, ^2^, ^3^ However, beginning in 2019, Wolf et al^3^ reported on the compassionate use of RAS/MAPK pathway inhibitors to downregulate hyperactivated signaling, and thus improve cardiac status through reduction in ventricular hypertrophy. In a larger case-control series, improved outcomes compared with standard therapy for these children have now been shown.^3^ In this case report, we highlight the additional complication confronting our patient, namely a large hemodynamically significant atrial septal defect (ASD) causing significant intracardiac shunting and contributing to pulmonary hypertension. To our knowledge, cardiac surgery in such an infant has not been reported to date; therefore, this report details the use of MEK inhibition with trametinib to stabilize heart failure related to RASopathy-associated HCM, and subsequent successful ASD closure resulting in significant clinical improvement and discharge home.

**Take-Home Message**

• Infants with severe presentations of NS-HCM and congenital heart defects can be medically stabilized with trametinib and then undergo successful structural heart surgery.

## Case Summary

**Figure 1:**
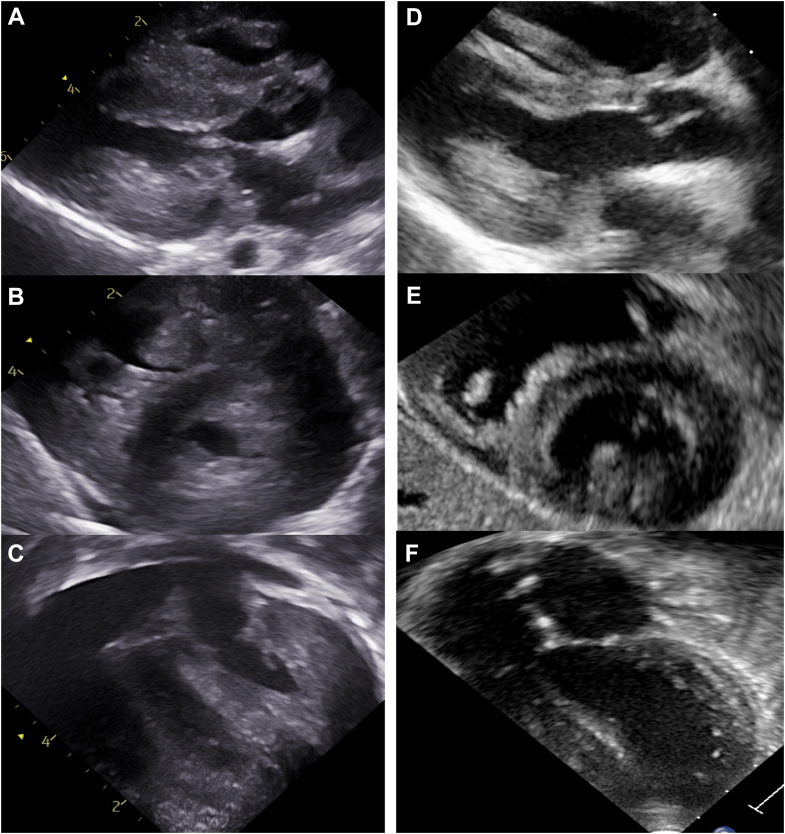
Echocardiographic Imaging at Admission and Discharge (A-C) Admission anatomy from parasternal long (A) and short (B) axis, and apical 4-chamber view (C). (D-F) Improvement in ventricular hypertrophy from corresponding views before discharge (after 9 months of MEK inhibition).

A 4-month-old boy was admitted to the hospital with failure to thrive and worsening respiratory distress. At admission, weight and height were both less than first percentile for age. Viral respiratory testing was positive for COVID-19 infection. Due to cardiac enlargement on chest radiography, an electrocardiogram was completed and showed northwest axis deviation with right ventricular hypertrophy and right atrial enlargement. Brain natriuretic peptide level (BNP) was 1,508 pg/mL. Echocardiography showed severe concentric left ventricular hypertrophy (LVH) without significant outflow tract obstruction. Additionally, a large, 12-mm, inferiorly located secundum ASD and smaller centrally located secundum ASD were noted. The pulmonary valve was mildly thickened but functioned well. Table 1 shows echocardiographic details, and Figure 1 shows images of the ventricular hypertrophy over time.

**Table 1:** Echocardiographic Findings During Treatment With Trametinib

	Admission	3 wk Postadmission: Start Trametinib	3 mo Postadmission	6 mo Postadmission	Discharge: 10 mo Postadmission
Age, mo	4	5	8	12	15
Weight, kg	5.1	5.4	6.7	8.7	9.5
Height, cm	56	56	61	67	70
IVS diameter, cm	1.35	1.42	0.82	0.7	0.65
IVS diameter, *z* score	5.9	6.1	3.3	2.3	1.8
LVEDd, cm	1.5	1.5	1.8	2	2.3
LVEDd, *z* score	−3.2	−3.3	−2.3	−2	−1.3
LVEDs, cm	0.6	0.75	1	1.2	1.5
LVEDs, *z* score	−6	−4.2	−2.6	−1.9	−0.7
LVFW diameter, cm	0.9	0.9	0.7	0.67	0.55
LVFW diameter, *z* score	5.5	5.5	3.9	3.1	2
LV mass index, g/height^2.7^	148	152	78	64	61
Tissue Doppler E′ velocity, MV annulus, cm/s	12	12	14	18	18
LV ejection fraction, %	86	79	74	69	69
LV outflow tract peak velocity, m/s	1.3	1.5	0.7	1.5	1.9
TR peak velocity, m/s	4.7	NR	3.5	3.8	2.8
PV peak velocity, m/s	3.2	3.2	2.5	2.8	1.5

IVS = interventricular septum; LV = left ventricle; LVEDd = LV end-diastolic dimension; LVEDs = LV end-systolic dimension; LVFW = LV free wall; MV = mitral valve; NR = not recorded; PV = pulmonary valve; TR = tricuspid regurgitation.

Due to the findings on electrocardiogram (extreme left axis deviation), short stature, mild dysmorphic features, and severe LVH, Noonan syndrome–associated hypertrophic cardiomyopathy (NS-HCM) was suspected. A genetics consult was obtained, and he was noted to have a small chin with a round face, low helices with mild posterior rotation of the ears, and a short neck. Molecular testing was sent (GeneDx rapid duo exome sequencing), which eventually returned with a heterozygous pathogenic variant in the *RIT1* gene, specifically c.246 T > G, p.F82 L. Given the severe cardiac findings, trametinib (MEK inhibition) was recommended due to reports of improvement in severe heart failure in these patients.^3^ Because this drug had never been used at our hospital before, there was some delay in initiation.

**Figure 2:**
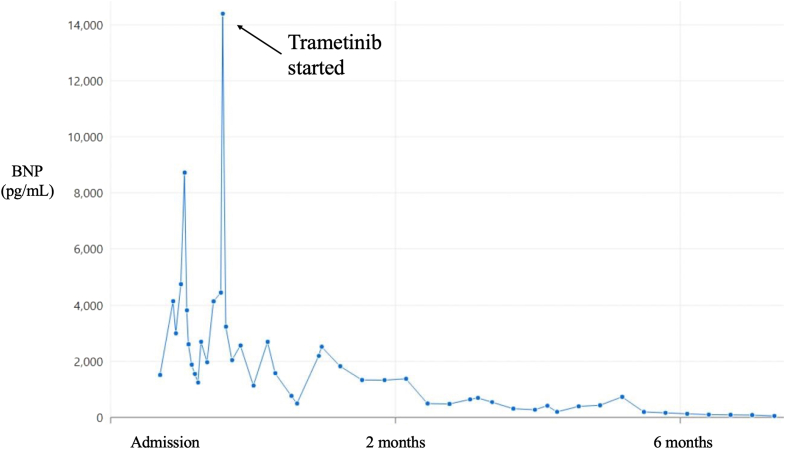
BNP Levels Over Time There was significant and enduring improvement in BNP, as a sign of improved heart failure status, with starting MEK inhibition. BNP = brain natriuretic peptide.

In the days after admission, despite supportive therapy and medications (remdesivir and dexamethasone) for COVID-19 pulmonary infection, there was significant clinical deterioration. He was transferred to the pediatric intensive care unit and subsequently intubated for respiratory failure. The patient was unstable, requiring sedation and paralysis to optimize ventilation, and serial BNP levels peaked at 14,396 pg/mL. Ventricular tachycardia occurred and self-resolved leading to beta-blocker initiation. At the time of peak BNP, trametinib (trametinib dimethyl sulfoxide 4.5 mg/90 mL oral solution, dose: 2.5 mL [0.125 mg] enterally daily) was obtained and started in the pediatric intensive care unit. In the week afterward, clinical stabilization occurred—respiratory status improved, paralytics were weaned, and ventilatory support lessened as well. BNP levels decreased to 1,131 and 491 pg/mL within 10 and 14 days, respectively, of starting trametinib and normalized with time (Figure 2). Echocardiography over the following weeks continued to show severe LVH with preserved systolic function and no left ventricular outflow tract obstruction. After multiple failed extubations, flexible bronchoscopy revealed tracheobronchomalacia but also severe left mainstem bronchus compression due to cardiomegaly. The decision was therefore made for tracheostomy and chronic mechanical ventilation.

Over the months following, the patient did not make typical progress because he continued to require high pressure and ventilation settings that precluded safe discharge. Echocardiography showed persistent significant atrial level shunting with right heart dilation and evidence of pulmonary hypertension (dilated pulmonary arteries, ventricular septal flattening, and systolic notching in the pulmonary artery Doppler signal). Significant diuresis was required to treat pulmonary overcirculation. Three diagnostic cardiac catheterizations were performed to delineate the physiology, with the initial study at 7 months of age demonstrating a high Q_p_/Q_s_ of 3.5 and mildly elevated pulmonary vascular resistance (pulmonary vascular resistance indexed [PVRI]: 3.5 Wood units [WU]/m^2^). Repeat catheterization at 10 months of age showed a more elevated PVRI of 7 WU/m^2^ with reduced Q_p_/Q_s_ of 1.1; however, a preceding bronchoscopy likely affected the findings. Medical treatment for pulmonary hypertension with sildenafil and bosentan was initiated. Finally, a third study 1 month later showed improved PVRI of 2.5 WU/m^2^ and a shunt fraction of 2.6. Left ventricular end-diastolic pressures and cardiac indexes were fairly consistent over time, with ranges 12 to 14 mm Hg and 2.3 to 3.5 L/min/m^2^, respectively. The defects were not amenable to closure in the catheterization laboratory due to the lack of an inferior rim, thus making seating a device safely across the septum unfeasible. After multiple discussions in cardiac conference, surgical closure was recommended.

The patient underwent surgery at 13 months of age. Trametinib was continued up until the day of surgery and then resumed on postoperative day 1. At surgery, the ASDs were closed using PhotoFix bovine (Artivion, Inc) pericardium in a standard fashion. A 4-mm fenestration was placed in the main patch due to concerns for pulmonary hypertension. Importantly, direct inspection of the heart during surgery confirmed the lack of significant hypertrophy, and standard cardioplegia dosing was used. Bypass time was 34 minutes, and cross clamp time was 22 minutes. There were no operative complications and intensive care unit recovery time was typical at 3 days’ duration.

**Figure 3:**
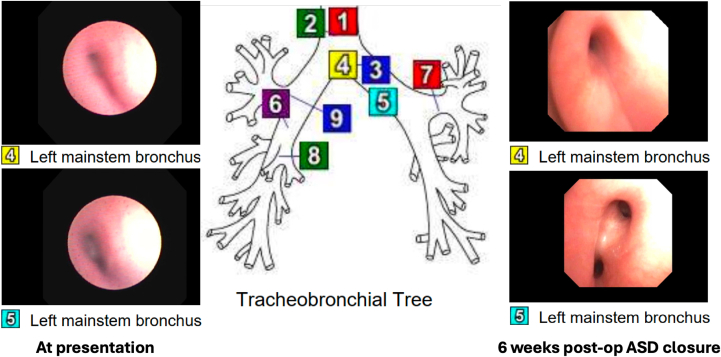
Flexible Bronchoscopy Evaluation of Left Mainstem Bronchus Left-sided images show proximal (upper) and distal (lower) left mainstem bronchus narrowing due to external compression at presentation. Right-sided images showed improvement in airway lumen size both proximally (upper) and distally (lower) before discharge (6 weeks after cardiac surgery).

Subsequently, the patient did well. Ventilator settings were now successfully weaned (at discharge, using synchronized intermittent mandatory ventilation/pressure support mode: with average inspiratory pressure of 14 mm Hg, end-expiratory pressure of 8 mm Hg, synchronized intermittent mandatory ventilation rate of 16 breaths/min, and 1 L oxygen to maintain saturations >94%), and diuretics were stopped. Echocardiography showed improved findings with less right heart dilation and improvement in LVH. Repeat bronchoscopy showed significant improvement in left bronchial compression (Figure 3). Echocardiography at time of discharge showed only mild LVH (see Table 1 and Figure 1, echocardiographic comparison). Discharge medications were sildenafil 1 mg/kg/dose 3 times per day, propranolol 2 mg/kg/d divided 3 times per day for history of ventricular tachycardia and optimization of left ventricular filling, and trametinib as previously mentioned. The patient was discharged home at 15 months of age after a 10-month hospital stay. Pulmonology will follow him closely over the next year and is hopeful for decannulation with time and growth. Despite the tracheostomy, the patient is thriving and meeting developmental milestones for age. Discharge weight was 9.5 kg (20th percentile, *z* = −0.7). We plan to continue trametinib through decannulation and complete improvement of LVH, noting reports of worsening LVH with weaning medication.^3^

## Discussion

**Figure 4:**
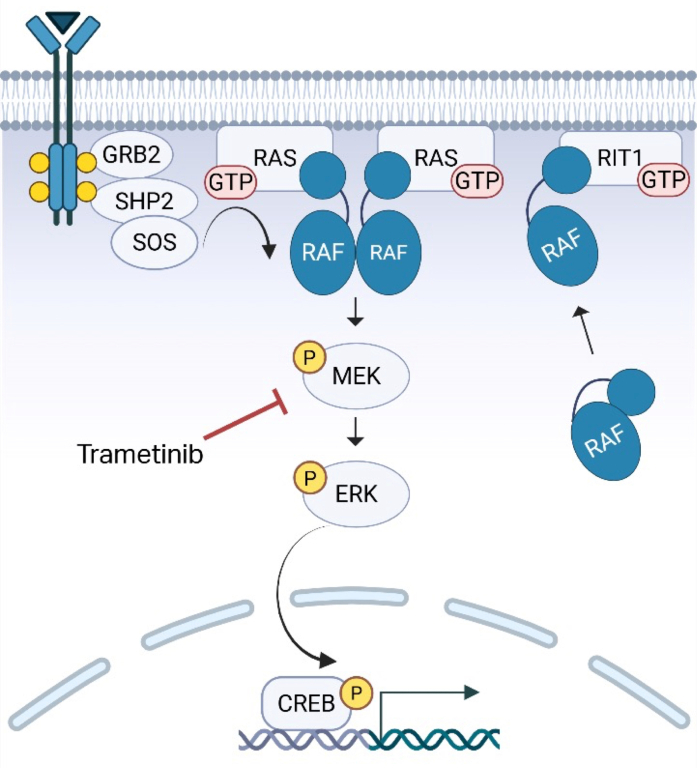
Role of RIT1 and MEK Inhibition in the RAS/MAPK Pathway Gain-of-function variants in the *RIT1* gene allow its GTP-loaded product to evade proteasomal degradation and accumulate at the plasma membrane. Cytosolic RAF is then recruited to the excess RIT1, and this local concentration of RAF is theorized to increase receptor tyrosine kinase signaling more robustly in the RAF/MEK/ERK signaling cascade. This leads to increased transcription of genes involved in cardiac cell growth and the development of hypertrophic cardiomyopathy. MEK inhibitors such as trametinib block this signaling cascade downstream, leading to attenuation of the excessive signaling.

Due to the important role of the RAS/MAPK pathway in many forms of cancer, chemotherapeutic medications aimed at downregulating this signaling pathway have been proposed and used over time.^4^, ^5^, ^6^, ^7^, ^8^ Specifically, MEK inhibitors, which attenuate abnormally upregulated signaling in the pathway due to gain-of-function mutations, have been used (Figure 4). As research deduced that RASopathies, including Noonan syndrome, are similar but milder gain of function germline variants compared with the oncologic processes,^9^ the use of MEK inhibitors to treat severe cardiac presentations in these patients eventually followed. Delineating the role of RAS signaling in the hypertrophic response of the cardiac ventricle to pressure overload, along with mice modeling showing reduction in hypertrophy with MEK inhibition, were key insights leading to eventual therapies in humans.^9^, ^10^, ^11^, ^12^ In 2019, Wolf et al^3^ first reported the use of the MEK inhibitor, trametinib, to treat 2 infants with severe HCM, with optimistic short-term results described. Now in 2025, authors from 23 centers worldwide reported retrospectively on 60 RASopathy HCM patients evenly split between standard of care heart failure therapies vs additional treatment with MEK inhibition.^3^ The MEK inhibitor–treated group showed significant improvement in risk of death, transplant, and/or need for cardiac surgery (left ventricular outflow tract resection):17% vs 87% risk in the untreated group (HR: 0.09; 95% CI: 0.04-0.25; *P* < 0.001). In taking care of our patient, it was heartening to read these reports and therefore anticipate our patient had a chance at a similarly improved outcome. It was even more gratifying to see the improvement actually occur as previously described—hemodynamic stability over the first week, then improvement in BNP over the weeks that followed, and finally decrease in ventricular hypertrophy requiring months of therapy.^3^^,^^9^ Side effects most commonly reported with trametinib include rash, fluid retention, diarrhea, fever, and constipation.^4^^,^^9^, ^10^, ^11^, ^12^ Our patient did develop significant eczema on medication; however, this was successfully managed with moisturizer and topical steroids. With medical stabilization, attention next turned to the remaining problem of a hemodynamically significant ASD.

Because device closure was not feasible due to the unfavorable septal anatomy (lack of inferior rim), surgical closure was the only repair option. Surgery for intracardiac defects in patients with hypertrophic cardiomyopathy is anticipated to be high risk due to the increased susceptibility of the hypertrophied ventricle to ischemic complications inherent to cardiopulmonary bypass and its recovery.^13^ Our patient also had pulmonary hypertension, a known risk factor for complications in this scenario.^13^, ^14^, ^15^ Finally, the risk inherent in any surgery in a NS-HCM patient taking trametinib was unknown. Therefore, our heart center was hesitant to recommend surgery, especially given the concern for unmasking more significant left ventricular diastolic dysfunction mitigated by an open ASD.^14^ Indeed, authors have reported on problematic ASD physiology in HCM patients, with fenestrated-type repairs proposed as a way to mitigate separately the effects of diastolic dysfunction and pulmonary hypertension.^15^ We therefore think that trametinib use for months before surgery was important to our patient's survival through surgery and to discharge. By the time of surgery, the extent of hypertrophy was much improved as was clinical status, in particular heart failure severity. To our knowledge, this is the first report of the use of trametinib as a “bridge” to congenital heart surgery, with a previous report of right ventricular outflow tract stenting being the only other report of any cardiac intervention in an infant on therapy.^16^ We hope our report will be useful to teams caring for these infants going forward.

## Conclusions

In an infant with NS-HCM presenting with cardiorespiratory failure, MEK inhibition (trametinib) was used to first stabilize the patient and ultimately allow for successful cardiac surgery (ASD closure). This first report on the clinical course illustrates steps to be taken in similar future patient encounters.

## Funding Support and Author Disclosures

The authors have reported that they have no relationships relevant to the contents of this paper to disclose.

**Visual Summary:** Timeline of the Case

Timeline	Events
Day 1	A 4-month-old was admitted with failure to thrive and respiratory distress. Diagnoses of severe hypertrophic cardiomyopathy and a large atrial septal defect were made and Noonan syndrome suspected.
Week 1	Worsening clinical status and heart failure despite supportive therapy, ultimately resulting in transfer to pediatric intensive care.
Week 2	A diagnosis of Noonan syndrome was confirmed. Tenuous clinical status despite optimization of medical therapy ultimately resulted in intubation and requirement for sedation and paralytic utilization.
Week 3	Trametinib, an MEK inhibitor that attenuates abnormal upregulated signaling in the RAS/MAPK pathway in Noonan syndrome, was started.
Week 4-5	Clinical stabilization occurred with weaning of ventilatory support and paralytics and decreasing markers of heart failure including brain natriuretic peptide levels.
Week 6	After multiple failed extubations, a bronchoscopy showed severe left mainstem bronchus compression due to cardiomegaly. A tracheostomy was performed.
Month 2	Despite improvement in heart failure and echocardiographic findings, including severity of ventricular hypertrophy, respiratory status and diuretic requirements remained high.
Month 3	Cardiac catheterization showed significant pulmonary overcirculation from the ASD in the context of mildly elevated ventricular filling pressures. Mildly elevated pulmonary vascular resistance was also present. The ASD was not amenable to device closure due to inadequate inferior rim. Surgical closure was not advised, and time and further medical therapy was recommended instead.
Months 4-5	Despite ongoing efforts at medical treatment and stabilization of respiratory status, the patient remained tenuous and unable to safely discharge. Echocardiograms continued to show improvement in ventricular hypertrophy but significant right heart dilation due to ASD shunting.
Months 6-7	Repeat heart catheterizations showed more elevated pulmonary vascular resistance requiring treatment with pulmonary vasodilators, and then improved findings similar to the initial catheterization. Eventually, a decision was made to proceed with surgical closure of the ASD.
Month 8	The patient underwent surgical closure of the ASD with a small fenestration left due to concerns for possible pulmonary hypertension exacerbation postoperatively. The surgery was uncomplicated, and intensive care postoperatively was typical and uneventful.
Month 9	Ventilator settings were now successfully weaned to reasonable home settings, and diuretics were stopped. Echocardiography at discharge showed only mild ventricular hypertrophy. Trametinib was continued at discharge and will be continued with close monitoring to facilitate progress toward decannulation.
Month 10	The patient was discharged home with cardiology and pulmonary follow-up. The patient is meeting age-appropriate developmental milestones and growing appropriately.

ASD = atrial septal defect.
